# Tobacco-Smoking, Alcohol-Drinking, and Betel-Quid-Chewing Behaviors: Development and Use of a Web-Based Survey System

**DOI:** 10.2196/mhealth.9783

**Published:** 2018-06-11

**Authors:** Kuo-Yao Hsu, Yun-Fang Tsai, Chu-Ching Huang, Wen-Ling Yeh, Kai-Ping Chang, Chen-Chun Lin, Ching-Yen Chen, Hsiu-Lan Lee

**Affiliations:** ^1^ Division of Orthopedic Sports Medicine Department of Orthopaedic Surgery Chang Gung Memorial Hospital at Linkou Tao-Yuan Taiwan; ^2^ College of Medicine Chang Gung University Tao-Yuan Taiwan; ^3^ School of Nursing College of Medicine Chang Gung University Tao-Yuan Taiwan; ^4^ Department of Nursing Chang Gung University of Science and Technology Tao-Yuan Taiwan; ^5^ Department of Psychiatry Chang Gung Memorial Hospital at Keelung Keelung Taiwan; ^6^ Division of Natural Science Center for General Education Chang Gung University Tao-Yuan Taiwan; ^7^ Department of Orthopedics Chang Gung Memorial Hospital at Linkou Tao-Yuan Taiwan; ^8^ Division of Head & Neck Surgery Department of Otolaryngology Chang Gung Memorial Hospital Tao-Yuan Taiwan; ^9^ Department of Hepato-Gastroenterology Chang Gung Memorial Hospital at Linkou Tao-Yuan Taiwan; ^10^ Department of Nursing Chang Gung Memorial Hospital at Linkou Tao-Yuan Taiwan

**Keywords:** tobacco smoking, alcohol drinking, betel-quid chewing, Web-based survey system

## Abstract

**Background:**

Smoking tobacco, drinking alcohol, and chewing betel quid are health-risk behaviors for several diseases, such as cancer, cardiovascular disease, and diabetes, with severe impacts on health. However, health care providers often have limited time to assess clients’ behaviors regarding smoking tobacco, drinking alcohol, and chewing betel quid and intervene, if needed.

**Objective:**

The objective of this study was to develop a Web-based survey system; determine the rates of tobacco-smoking, alcohol-drinking, and betel-quid-chewing behaviors; and estimate the efficiency of the system (time to complete the survey).

**Methods:**

Patients and their family members or friends were recruited from gastrointestinal medical–surgical, otolaryngology, orthopedics, and rehabilitation clinics or wards at a medical center in northern Taiwan. Data for this descriptive, cross-sectional study were extracted from a large series of research studies. A Web-based survey system was developed using a Linux, Apache, MySQL, PHP stack solution. The Web survey was set up to include four questionnaires: the Chinese-version Fagerstrom Tolerance Questionnaire, the Chinese-version Alcohol Use Disorders Identification Test, the Betel Nut Dependency Scale, and a sociodemographic form with several chronic diseases. After the participants completed the survey, the system automatically calculated their score, categorized their risk level for each behavior, and immediately presented and explained their results. The system also recorded the time each participant took to complete the survey.

**Results:**

Of 782 patient participants, 29.6% were addicted to nicotine, 13.3% were hazardous, harmful, or dependent alcohol drinkers, and 1.5% were dependent on chewing betel quid. Of 425 family or friend participants, 19.8% were addicted to nicotine, 5.6% were hazardous, harmful, or dependent alcohol drinkers, and 0.9% were dependent on chewing betel quid. Regarding the mean time to complete the survey, patients took 7.9 minutes (SD 3.0; range 3-20) and family members or friends took 7.7 minutes (SD 2.8; range 3-18). Most of the participants completed the survey within 5-10 minutes.

**Conclusions:**

The Web-based survey was easy to self-administer. Health care providers can use this Web-based survey system to save time in assessing these risk behaviors in clinical settings. All smokers had mild-to-severe nicotine addiction, and 5.6%-12.3% of patients and their family members or friends were at risk of alcohol dependence. Considering that these three behaviors, particularly in combination, dramatically increase the risk of esophageal cancer, appropriate and convenient interventions are necessary for preserving public health in Taiwan.

## Introduction

Tobacco smoking has devastating global health, social, environmental, and economic consequences [1]. Each year, >7 million people worldwide die from tobacco use [2], with over 80% of deaths occurring in low- or middle-income countries [3]. On average, tobacco users lose 15 years of life [4]. Up to 50% of all tobacco users die of tobacco-related causes, including heart disease, cancer, diabetes, and lung disease [5]. Illnesses caused by tobacco use also contribute to poverty by increasing health care spending for individuals and families [1]. In addition, tobacco waste contains over 7000 toxic chemicals, including human carcinogens. Tobacco smoke emissions also contribute thousands of tons of human carcinogens, toxicants, and greenhouse gases to the environment [1]. Moreover, tobacco use imposes a substantial economic burden globally. Smoking-attributable costs, both direct (eg, the cost incurred by the utilization of health care services) and indirect (eg, any additional cost incurred as a result of the utilization of health care services), are estimated to be US $ 1400 billion or 1.8% of the global gross domestic product [6].

Excessive alcohol use has been associated with a large variety of health, social, and legal problems [7]. In 2012, approximately 3.3 million deaths, or 5.9% of all deaths worldwide, were attributable to alcohol consumption [8]. Alcohol consumption is associated with an increased risk of over 200 diseases and injuries [8]; it has also been associated with mental and behavioral disorders, including alcohol dependence, liver cirrhosis, some cancers, and cardiovascular diseases, as well as injuries from violence and traffic accidents [8].

Betel-quid chewing is a popular habit in south and Southeast Asia [9]; it has a carcinogenic effect and is associated with obesity, hypertriglyceridemia, hyperglycemia, metabolic syndrome, cardiovascular disease, hepatic dysfunction, liver cirrhosis, and liver cancer [10-12].

In countries such as Taiwan, where people commonly smoke, drink alcohol, and chew betel quid, esophageal cancer is prevalent [13]. Indeed, the concurrent use of alcohol and tobacco leads to a higher risk of esophageal cancer (odds ratio [OR] 8), and the addition of betel-quid chewing can increase the odds ratio to 195.6 (95% CI 64.0-894.2) [14,15].

In Taiwan, the smoking rate of adults dropped from 32.5% in 1990 to 15.3% in 2016, but there were still 3.13 million tobacco smokers (individuals who smoked >100 cigarettes to date and had smoked in the last 30 days) [16]. Alcohol, which is legally accessible in Taiwan, plays an important role in Chinese culture as it is viewed as an acceptable drink to relieve stress and enhance social interaction [17]. Consequently, drinking problems are easily ignored. In Taiwan’s general hospitals, the prevalence of patients’ alcohol-drinking problems (Alcohol Use Disorders Identification Test, AUDIT, score ≥8) ranges from 5.7% to 19.2% due to different settings (wards versus clinics) selected [18,19]. In addition, chewing betel quid is a part of traditional Chinese culture, and it is often offered during many social occasions in Taiwan [20]. A national survey conducted in the last 6 months of 2013 found that the rates of adult smoking (individuals smoking >100 cigarettes) and alcohol drinking (individuals drinking alcohol liquor not included in cooking) were 22.6% and 39.2%, respectively; in both the previous year and previous 6 months, the rate of chewing betel quid was 6.4% [21]. These statistics indicate that smoking, drinking alcohol, and chewing betel quid are troubling health-risk behavior indicators in Taiwan.

Considering that individuals who smoke tobacco, drink alcohol, and chew betel quid are likely to suffer adverse effects on their health (eg, cancer, cardiovascular disease, and diabetes), they will need to seek health care. Thus, health care providers are in a unique position to both identify and treat patients with these unhealthy behaviors. However, limited time often presents providers with a barrier to implement both appropriate assessment and intervention strategies [22,23]. This barrier may be overcome by using a Web-based system. Indeed, Web-based systems have been found to arouse participants’ interests about their own health, maintain privacy, increase reasonable response rates, and save costs [24]. Unfortunately, most Web-based systems are available only for alcohol screening and interventions [25], and no Web-based system combines tobacco smoking, alcohol drinking, and betel-quid chewing. To address this knowledge gap, we undertook this study to develop a Web-based survey system and determine the rates of tobacco-smoking, alcohol-drinking, and betel-quid-chewing behaviors in patients and their family members or friends recruited from a medical center in Taiwan. We also estimated the efficiency of this system by calculating the time to complete the survey.

## Methods

### Design

This descriptive cross-sectional study was a part of an extensive research series to promote healthy lifestyle behaviors for the general population. Data were collected using a Web-based survey from 2015 to 2016.

### Sample and Setting

The sample was recruited by a trained research assistant (RA) from the waiting areas of gastrointestinal medical–surgical, otolaryngology, orthopedics, and rehabilitation clinics/wards at a medical center in northern Taiwan. These clinics and wards were chosen because most patients with alcohol or smoking problems in Taiwan are seen here [26]. Patients were included if they met the following criteria: (1) ≥20 years old, (2) outpatients or inpatients in the above clinics or wards, (3) had mobile phones or email access, and (4) able to read Chinese. Similar inclusion criteria were used for patients’ family members, partners, and friends.

Patients were approached by the RA who told them that our research team was interested in developing a Web-based substance-use intervention system and would like their participation for at least 20 minutes to help develop the new system. After obtaining written consent from patients, family members, and friends to participate, the RA screened each one for both inclusion and exclusion criteria. Of the 1400 patients approached, only 1210 agreed to participate. Those who refused to participate gave lack of time as the main reason for refusing. Of the patients who agreed to participate, 3 did not meet the inclusion criteria; thus, 1207 completed the survey. No participants dropped out of this study.

### The Web-Based Survey System

The system was built using a Linux, Apache, MySQL, PHP stack solution. The Linux operation system was hosted on a lightweight Dell server (with two Intel 3.0-GHz CPU processors and 8G RAM) to provide the survey via the internet. The Apache web server system runs on the Dell server. To address the gap of no appropriate software packages available to set up a Web-based survey system, we developed our system from scratch using *PHP: hypertext preprocessor* (PHP) language, a well-known, reliable server-site technology. Within the survey system, we set up four questionnaires or scales: the Chinese-version Fagerstrom Tolerance Questionnaire (C-FTQ) [27], the Chinese-version AUDIT (C-AUDIT) [26], the Betel Nut Dependency Scale (BNDS) [28], and a sociodemographic form. The C-FTQ was chosen because it has been used as a screening tool for tobacco control by Taiwan’s Health Promotion Administration, Ministry of Health and Welfare [27]. The C-AUDIT was translated and validated by our research team [26] from the World Health Organization (WHO)-developed AUDIT, a commonly used and widely translated tool for screening alcohol problems [29]. The BNDS, which is the only available screening tool for betel-nut dependence in the general population of Taiwan, has undergone comprehensive psychometric testing [28]. After participants completed the survey, their scores were automatically calculated, their risk behaviors were categorized (from no risk to high risk, depending on each questionnaire’s definitions; see next section), and their results were immediately presented with explanations. The system also recorded the time required for each participant to complete the survey.

Before using the Web-based survey system in clinical settings, we tested its stability and accuracy. Testing involved the following: setting up an account and password to access the survey; answering questions; and checking scores, categories, and explanations. A technician and RAs in our lab tested the system more than 10,000 times over 3 months. In addition, we retrieved all data, entered it into statistical software, and regularly calculated the total score for each tool to ensure the accuracy of the process. No mistakes were detected.

### Study Variables

These scales were used to collect data on participants’ physical dependence on nicotine, alcohol drinking-related behaviors, betel-quid dependency, and sociodemographic characteristics plus several chronic diseases.

Physical dependence on nicotine in the previous year was measured using the 7-item C-FTQ. The first item asks, “Did you smoke in the past year?” If the participant answers “no,” he or she skips the remaining 6 items and the score is 0. If the participant answers “yes” to the first item, he/she answers the remaining items. The total C-FTQ score represents the sum of items 2-7; possible score range 0-10. A score of 0 indicates no smoking behavior. Summed scores ≤3 indicate mild nicotine addiction, 4-6 indicates moderate addiction, and 7-10 indicates high addiction [27]. The validity and reliability of the FTQ [30,31] and C-FTQ [32] were acceptable. The internal consistency of the C-FTQ in this study was 0.76.

Alcohol drinking-related behaviors in the previous year were measured using the 10-item C-AUDIT. Each item was scored on a 4-point Likert scale from 0 to 1, with total scores ranging from 0 to 40. Scores 0-7 indicate low-risk drinking, 8-15 indicate hazardous drinking, 16-19 indicate harmful drinking, and ≥20 indicate dependence in drinking [29]. The validity and reliability of the AUDIT [29] and C-AUDIT [26] were acceptable. The internal consistency of the C-AUDIT in this study was 0.83.

Betel-quid dependency in the previous year was measured using the 11-item BNDS. Items were scored on a 4-point Likert scale (1=*totally agree*, 2=*agree*, 3=*disagree*, 4=*totally disagree*). Item scores were summed for a total score, with a possible range of 11-44; scores ≥24 indicate a tendency toward betel-quid dependency [28]. The validity and reliability of the BNDS were acceptable [28]. The internal consistency of the BNDS in this study was 0.96.

Sociodemographic characteristics (age, gender, education level, marital status, number of children) were measured using a sociodemographic form. Chronic illnesses (arthritis, cancer, cardiovascular disease, cataract, diabetes, digestive system disease, epilepsy, gout, hyperlipidemia, hypertension, kidney disease, liver disease, stroke, urinary tract disease) were measured using a chronic illness checklist.

### Data Collection

After screening participants for study criteria, the RA provided each one a sealed envelope containing a login account and password. The RA also provided written instructions for accessing the web system. The participants read these instructions by themselves. The RA then helped the participants find a seat in the waiting area, gave them a laptop computer, and let them complete the survey alone. The participants connected to the internet via the participating hospital’s free Wi-Fi. If the free Wi-Fi was not accessible in the waiting area, the participants connected to a 4G wireless network paid by researchers. After the participants finished the online survey, it automatically provided their score on each questionnaire with explanations of the scores. The system also reminded the participants to discuss any health concerns with their health care providers. After the participants finished the survey, they caught the RA’s attention to return the laptop computer. The RA stayed in the waiting area in case the participants needed any help. In addition, the RA provided information to the participants about tobacco smoking, alcohol drinking, and betel-quid chewing, if they needed it, after completing the survey.

### Ethical Considerations

After the institutional review board of Chang Gung Memorial Hospital approved the study, the RA approached patients, their family members, or friends in the wards or clinic waiting areas. The RA described the study purpose and procedure, the required time commitment, confidentiality, and participants’ rights not to participate or to withdraw from the study at any time and obtained their written consent to participate. The participants received a small gift (approximately US $ 3) for their participation.

### Data Analysis

Sociodemographic and questionnaire data were analyzed by descriptive statistics (mean, standard deviation, and frequency [percentage]) using SPSS, version 22.

## Results

The average age of the 782 patients who participated in this study was 35.9 years (SD 11.9; range 20-83). Most of the patients were male (533/782, 68.2%), had graduated from a college or university (435/782, 55.6%), were single (408/782, 52.2%), and sought treatment in orthopedics clinics or wards (492/782, 62.9%). For details, see Table 1. Patients’ top three chronic diseases were liver diseases (77/782, 9.8%), hypertension (71/782, 9.1%), and diabetes (31/782, 4.0%) (see Table 2 for details).

The 425 patients’ family members and friends in our sample were on average 35.3 years old (SD 10.8; range 20-79). Most of them were male (216/425, 50.8%) and had graduated from a college or university (250/425, 58.8%). Approximately half of them were single (207/425, 48.7%) or married (206/425, 48.5%). For details, see Table 1. For these family members and friends, the top three common chronic diseases were liver diseases (19/425, 4.5%), hypertension (19/425, 4.5%), and diabetes (9/425, 2.1%) (see Table 2 for details).

**Table 1:** Participants’ demographic characteristics.

Variable		Patients	Family members and friends
**Gender, n (%)**			
	Male	533 (68.2)	216 (50.8)
	Female	249 (31.7)	209 (49.2)
Age (years), mean (SD)		35.9 (11.9)	35.3 (10.8)
**Visit clinic or ward, n (%)**			
	Orthopedics	492 (62.7)	232 (54.5)
	Otolaryngology	188 (23.9)	114 (26.8)
	Gastroenterology	99 (12.6)	75 (17.6)
	Rehabilitation medicine	3 (0.4)	4 (0.9)
**Education level, n (%)**			
	Illiterate	4 (0.5)	4 (0.9)
	Primary school	20 (2.6)	2 (0.5)
	Junior high school	46 (5.9)	18 (4.2)
	Senior high school	165 (21.1)	113 (26.6)
	College or university	435 (55.6)	250 (58.8)
	Master’s degree or above	112 (14.3)	38 (8.9)
**Marital status, n (%)**			
	Single	408 (52.2)	207 (48.7)
	Married	354 (45.3)	206 (48.5)
	Divorced	17 (2.2)	11 (2.6)
	Widowed	3 (0.4)	1 (0.2)
Number of children, mean (SD)		0.9 (1.2)	0.9 (1.2)

**Table 2:** Participants’ chronic illnesses.

Illness	Patients, n (%)	Family members and friends, n (%)
Liver disease	77 (9.8)	19 (4.5)
Hypertension	71 (9.1)	19 (4.5)
Diabetes	31 (4.0)	9 (2.1)
Arthritis	29 (3.7)	2 (0.5)
Cancer	23 (2.9)	1 (0.2)
Digestive system disease	15 (1.9)	8 (1.9)
Cardiovascular disease	15 (1.9)	7 (1.6)
Hyperlipidemia	8 (1.0)	5 (1.2)
Gout	8 (1.0)	4 (0.9)
Urinary tract disease	7 (0.9)	2 (0.5)
Kidney disease	6 (0.8)	2 (0.5)
Cataract	2 (0.3)	0 (0.0)
Epilepsy	1 (0.1)	1 (0.2)
Stroke	1 (0.1)	0 (0.0)

**Table 3:** Participants’ smoking, drinking, and betel-quid-chewing scores.

Variable		Patients	Family members and friends
C-FTQ^a^ score, mean (SD)		1.1 (2.2)	0.7 (1.9)
**C-FTQ addiction level, n (%)**			
	No addiction	550 (70.3)	341 (80.2)
	Mild addiction	111 (14.2)	44 (10.4)
	Moderate addiction	84 (10.7)	25 (5.9)
	High addiction	37 (4.7)	15 (3.5)
C-AUDIT^b^ score, mean (SD)		2.8 (4.5)	1.7 (3.2)
**C-AUDIT level, n (%)**			
	Low-risk drinker	678 (86.7)	401 (94.4)
	Hazardous drinker	78 (10.0)	18 (4.2)
	Harmful drinker	18 (2.3)	6 (1.4)
	Dependent drinker	8 (1.0)	0 (0.0)
BNDS^c^, mean (SD)		11.6 (2.9)	11.4 (2.6)
**BNDS level, n (%)**			
	No potential addiction	770 (98.5)	421 (99.1)
	Potential addiction	12 (1.5)	4 (0.9)

^a^C-FTQ: Chinese-version Fagerstrom Tolerance Questionnaire.

^b^C-AUDIT: Chinese-version Alcohol Use Disorders Identification Test.

^c^BNDS: Betel Nut Dependency Scale.

Patients’ mean C-FTQ, C-AUDIT, and BNDS scores were 1.1 (SD 2.2), 2.8 (SD 4.5), and 11.6 (SD 2.9), respectively. Among patients, 29.7% (232/782) were mildly to highly addicted to nicotine; 13.3% (104/782) were hazardous, harmful, or dependent drinkers; and 1.5% (12/782) were betel-quid dependent. Family members’ and friends’ mean C-FTQ, C-AUDIT, and BNDS scores were 0.7 (SD 1.9), 1.7 (SD 3.2), and 11.4 (SD 2.6), respectively. Among family members and friends, 19.8% (84/425) were addicted to nicotine, 5.6% (24/425) were hazardous or harmful drinkers, and 0.9% (4/425) were betel-quid dependent. For details, see Table 3.

**Table 4:** Participants’ smoking, drinking, and betel-quid-chewing behaviors.

Risk behavior	Patients, n (%)	Family members and friends, n (%)
None	256 (32.7)	179 (42.1)
Smoking only	35 (4.5)	20 (4.7)
Drinking only	287 (36.7)	161 (37.9)
Betel-quid chewing only	0 (0.0)	0 (0.0)
Smoking and drinking	131 (16.8)	44 (10.4)
Smoking and betel-quid chewing	9 (1.2)	4 (0.09)
Drinking and betel-quid chewing	7 (0.09)	1 (0.02)
Smoking and drinking and betel-quid chewing	57 (7.3)	16 (3.8)

Only 32.7% (256/782) of patients did not smoke, drink alcohol, and chew betel quid in the previous year. Over 60% (482/782, 61.6%) of patients drank alcohol in the past year (including drinking only, smoking and drinking, drinking and betel-quid chewing, and smoking and drinking and betel-quid chewing). Similarly, only 42.1% (179/425) of family members and friends did not smoke, drink alcohol, and chew betel quid in the previous year. Over 52% (222/425, 52.2%) of family members and friends drank alcohol in the past year. For details, see Table 4.

Regarding the mean time to complete the survey, patients needed 7.9 minutes (SD 3.0; range 3-20) and family members or friends needed 7.7 minutes (SD 2.8; range 3-18). Most patients completed the survey within 5-10 minutes (656/782, 83.9%), and most family members or friends completed it within 5-10 minutes (363/425, 81.7%).

## Discussion

### Web-Based Survey System

Our Web-based survey system to assess behaviors regarding drinking alcohol, smoking tobacco, and chewing betel quid contributes to the literature on health-risk behaviors by providing a survey that patients can complete easily, quickly, and entirely. Most participants completed it within 5-10 minutes and perceived it as being easy to self-administer. The survey was designed to ensure that the participants could not skip any item, thus avoiding missing data, by requiring that each item be filled in before moving to the next page. Furthermore, our data on the internal consistency of the Web-versions of the C-FTQ, C-AUDIT, and BNDS were all acceptable. Unlike other assessment tools, such as the Kihon checklist [33], which must be administered by health care providers, using this survey can save time for providers to assess these risk behaviors in clinical settings. Moreover, the survey results provide client data for health-promotion strategies on smoking tobacco, drinking alcohol, and chewing betel quid.

Regarding internet access for the online survey, we initially used the participating hospital’s new, free Wi-Fi for visitors, but this system was unstable. We did not want to ask the participants to use their own smart phone, if the free Wi-Fi did not work, because they might have worried about increasing their internet service fee and refused to participate in our study. Therefore, we provided a laptop for the participants and allowed them to use either free Wi-Fi or a researcher-paid 4G network to complete the survey. Currently, the hospital’s free Wi-Fi system for visitors is stable. Once our research on the Web-based survey and its efficacy is published, we plan to add a quick response (QR) code system to the survey. Patients and family members can use their own smart phone to scan the QR code and fill out the survey. This change will increase the accessibility of the online survey system in clinical settings. After the present Web-based survey system matures, its use may be expanded to include the public setting for assessing health behaviors in the general population.

### Smoking, Drinking, and Betel-Quid-Chewing Behaviors

Approximately 30% of patients and 20% of family members and friends smoked in the past year, but all of them were categorized as having mild-to-severe nicotine addiction. These prevalence rates for tobacco smoking are in line with the 2010 WHO global prevalence of 22.1% [34] but exceed Taiwan’s national rate of 15.3% established by the Adult Smoking Behavior Surveillance System in 2016 [13]. Given that tobacco smoking has a negative impact on health, it is essential to decrease smoking behaviors. To assist governments worldwide in reducing both tobacco demand and use**,** in 2008, the WHO introduced a package of six evidence-based strategies [34]. These strategies, known as the MPOWER package, include Monitoring tobacco use and prevention policies; Protecting people from tobacco smoke; Offering help to quit tobacco use; Warning about the dangers of tobacco; Enforcing bans on tobacco advertising, promotion, and sponsorship; and Raising taxes on tobacco [34]. Among these strategies, those that could be implemented in clinical settings include establishing smoke-free buildings and grounds, offering smoking-cessation programs, providing warning labels on tobacco packages, and enforcing bans on tobacco promotion.

Among our participants, over 60% of patients and over 52% of family members or friends drank alcohol in the past year, and 13.3% of patients and 5.6% of family members or friends were categorized as hazardous, harmful, or dependent drinkers. The prevalence for our patient sample is at the low end of the range reported for alcohol-drinking problems in general hospitals in western countries, that is, 12% to 26%, due to different assessment methods and units selected [35,36]. However, no information is available on studies conducted in western countries for the prevalence of alcohol-drinking problems among family members of these patients. Our patient sample’s prevalence of hazardous, harmful, or dependent drinkers is consistent with that of patients’ alcohol-drinking problems in Taiwan’s general hospitals, that is, 5.7% to 19.2%, depending on the unit selected [18,19]. However, our finding on the prevalence of patients’ family members’/friends’ hazardous/harmful/dependent drinking (5.6%) is much lower than that of hazardous alcohol-drinking problems among family members of patients with alcohol-drinking problems in Taiwan (13.3%) [37]. The difference between the findings of these studies may be because our sample of family members or friends included approximately 25% friends (105/425). Indeed, individuals with a family history of alcoholism have been linked to a greater risk of developing alcoholism than those without such a history [38].

To manage the drinking behavior of individuals with hazardous and harmful drinking, a brief intervention [18] has proven to be effective in a Taiwanese hospital patient population [18,22]. This brief intervention is offered to problem drinkers at four levels, based on their AUDIT score [22]. The first level, for low-risk drinkers, is to provide alcohol education. The second level, for hazardous drinkers, is to give simple advice (including giving feedback, providing information, establishing goals to change drinking behavior, giving advice on drinking limits, reviewing the amount of alcohol in standard drinks, and concluding with encouragement). The third intervention level, for harmful drinkers, is to provide simple advice plus brief counseling and continued monitoring. The fourth level, for dependent drinkers, is to refer them to a specialist for diagnostic evaluation and treatment [22]. This brief intervention should be considered as a strategy to decrease our participants’ alcohol drinking behaviors.

The prevalence of betel-quid dependency was low in both patients and family members or friends. Currently, no effective interventions for betel-quid chewing are available. Our findings show that no participants chewed betel quid alone; they only used it in combination with smoking or drinking alcohol. This finding implies that interventions against betel-quid chewing should be combined with antismoking or alcohol-drinking interventions.

### Strengths and Limitations

Our study fills a knowledge gap by both developing and applying a convenient Web-based survey system to assess behaviors regarding smoking tobacco, drinking alcohol, and chewing betel quid. Our results demonstrate that the system is easy to use for both patients and their family members or friends. However, this study had four limitations. First, the study population was a convenient sample recruited from one hospital in northern Taiwan. Second, participants were included only if they had mobile phones or email access. In other words, they had some experience using Web-based systems. Third, participants may have been hindered in expressing whether the survey was difficult when asked in person by the RA. Fourth, we did not follow-up with participants to determine if they had discussed their survey results with their health care providers because this follow-up was beyond the scope of our study. For the same reason, we did not survey physicians regarding both their knowledge of the widely used tobacco-smoking, alcohol-drinking, and betel-quid-chewing scales used in our survey and their readiness to assess their clients for these health-risk behaviors. Further studies may consider using a random sampling strategy; expanding the study criteria to include people naïve to computers, communication, and consumer products; following up with both patients and their health care providers on the application of survey results; and including an item at the end of the survey to ask anonymously about the difficulty in completing the survey.

### Conclusions

Smoking, drinking alcohol, and chewing betel quid are risk behaviors with severe impacts on health. However, health care providers often have little or limited time to evaluate their clients for these risk behaviors. Using our newly developed Web-based survey system can save clinicians’ time for assessing patients and offering participants data about their health behaviors. We also found that all smokers had a mild-to-severe nicotine addiction, and both patients and their family members or friends had a high prevalence of drinking alcohol in the past year. Furthermore, 5.6%-12.3% of patients and their family members or friends were at risk of alcohol dependence. Since these three behaviors, particularly in combination, dramatically increase the risk of esophageal cancer, appropriate and convenient interventions are necessary for maintaining public health in Taiwan.

## Abbreviations

AUDIT: Alcohol Use Disorders Identification Test
BNDS: Betel Nut Dependency Scale
C-AUDIT: Chinese-version Alcohol Use Disorders Identification Test
C-FTQ: Chinese-version Fagerstrom Tolerance Questionnaire
FTQ: Fagerstrom Tolerance Questionnaire
RA: research assistant
WHO: World Health Organization
